# Frailty, gaps in care coordination, and preventable adverse events

**DOI:** 10.1186/s12877-022-03164-7

**Published:** 2022-06-02

**Authors:** Oluwasegun P. Akinyelure, Calvin L. Colvin, Madeline R. Sterling, Monika M. Safford, Paul Muntner, Lisandro D. Colantonio, Lisa M. Kern

**Affiliations:** 1 Department of Epidemiology, University of Alabama at Birmingham, Birmingham, AL USA; 2 Department of Medicine, Weill Cornell Medicine, 420 East 70th Street, Box 331, New York, NY 10021 USA

**Keywords:** Frailty, Gaps in care coordination, Adverse events, Preventable emergency department visit, Preventable hospitalization

## Abstract

**Background:**

Older US adults often receive care from multiple ambulatory providers. Seeing multiple providers may be clinically appropriate but creates challenges for communication. Whether frailty is a risk factor for gaps in communication among older adults and subsequent preventable adverse events is unknown.

**Methods:**

We conducted a cross-sectional analysis of community-dwelling US adults ≥ 65 years of age in the REasons for Geographic And Racial Differences in Stroke (REGARDS) study who attended an in-home study examination in 2013–2016 and completed a survey on experiences with healthcare in 2017–2018 (*n* = 5,024). Using 5 frailty indicators (low body mass index, exhaustion, slow walk, weakness, and history of falls), we characterized participants into 3 mutually exclusive groups: not frail (0 indicators), intermediate-frail (1–2 indicators), and frail (3–5 indicators). We used survey data on self-reported gaps in care coordination and self-reported adverse events that participants attributed to poor communication among providers (a drug-drug interaction, repeat testing, an emergency department visit, or a hospital admission).

**Results:**

Overall, 2,398 (47.7%) participants were not frail, 2,436 (48.5%) were intermediate-frail, and 190 (3.8%) were frail. The prevalence of any gap in care coordination was 37.0%, 40.8%, and 51.1% among participants who were not frail, intermediate-frail and frail, respectively. The adjusted prevalence ratio (PR) for any gap in care coordination among intermediate-frail and frail versus not frail participants was 1.09 (95% confidence interval [95%CI] 1.02–1.18) and 1.34 (95%CI 1.15–1.56), respectively. The prevalence of any preventable adverse event was 7.0%, 11.3% and 20.0% among participants who were not frail, intermediate-frail and frail, respectively. The adjusted PR for any preventable adverse event among those who were intermediate-frail and frail versus not frail was 1.47 (95%CI 1.22–1.77) and 2.24 (95%CI 1.60–3.14), respectively.

**Conclusion:**

Among older adults, frailty is associated with an increased prevalence for self-reported gaps in care coordination and preventable adverse events. Targeted interventions to address patient-reported concerns regarding care coordination among intermediate-frail and frail older adults may be warranted.

**Supplementary Information:**

The online version contains supplementary material available at 10.1186/s12877-022-03164-7.

## Background

Frailty, a syndrome characterized by a decline in the overall function of multiple body systems and decreased resistance to stressors [1], is a common condition among older community-dwelling US adults [2–5]. Among older adults, frailty has been associated with increased morbidity and mortality, risk for hospitalization, and healthcare utilization and costs [1, 6–9]. Older adults who are frail often have multiple chronic conditions and complex healthcare needs, which may be managed by multiple healthcare providers [2, 6, 10–14]. Having multiple healthcare providers may be clinically appropriate, but may pose challenges for adequate communication among those providers [15]. As providers do not consistently share information about patients they have in common [15], gaps in care coordination may occur.

Previous research has found that patient-reported gaps in care coordination are common, with 38% of adults 65 years and older reporting a problem with the coordination of their healthcare in the previous 6 months [16]. These self-reported problems in care coordination were associated with self-reported preventable adverse events, or events that individuals reported could have been prevented with better care coordination, including drug-drug interactions, repeat testing, preventable emergency department visits, and preventable hospital admissions [16]. However, it is not known whether frail older adults are more likely to experience gaps in care coordination and associated adverse events versus their counterparts who are not frail. Also, a prior study has shown that older adults who report gaps in care coordination are more likely to have adverse events [16], but it is not known if this is generalizable to frail older adults. If frailty is associated with gaps in care coordination and preventable adverse events, this would be relevant to know, because such suboptimal healthcare for this vulnerable population is potentially modifiable.

The goal of the current study was to evaluate the association of frailty with gaps in care coordination and preventable adverse events among older US adults. To accomplish this goal, we analyzed data from the REasons for Geographic And Racial Differences in Stroke (REGARDS) study.

## Methods

The REGARDS study enrolled a population-based cohort of 30,239 Black and White adults aged ≥ 45 years from the 48 contiguous US states and the District of Columbia between 2003 and 2007. Blacks and individuals living in the southeastern US were oversampled, because the study was designed to elucidate reasons for racial and geographic differences in stroke mortality. The study details has been previously published [17]. The REGARDS study was approved by the institutional review boards of the participating centers, and all participants provided written informed consent. The current analysis was approved by the institutional review board at the University of Alabama at Birmingham.

For the current study, we restricted the analysis to participants who completed a second in-home examination and a computer assisted telephone interview (CATI) between 2013 and 2016 and were aged ≥ 65 years (Supplemental Fig. 1). We further restricted the analysis to participants not living in a nursing home who completed a survey on experiences with healthcare between August 2017 and November 2018 and reported having a regular healthcare provider, had > 1 visit and > 1 provider in the past 12 months (thus being at risk for problems with care coordination), and saw the regular provider in the prior 6 months (consistent with the look-back period for questions regarding perceived gaps in care coordination). We excluded participants with cognitive impairment at the time of the survey on healthcare experiences, defined as having a Six-Item Screener score (SIS) [18] ≤ 4 using the most recent assessment available on or before the survey, or by having 2 consecutive SIS scores ≤ 4 any time on or before the completion of the survey. Finally, we restricted the analysis to participants with valid data on ≥ 3 of the 5 indicators of frailty included in the analysis as described below. Our final analytic sample included 5,024 participants.

### Frailty indicators

We analyzed 5 frailty indicators assessed at the second REGARDS CATI and in-home examination: low body mass index (BMI), exhaustion, slow walk, weakness, and history of falls. These indicators were adapted from the definition of frailty by Fried et. al., using data available in the REGARDS study (see Supplemental Table 1) [2, 19]. Fried et al. [2] used data on unintentional weight loss (lost more than 10 pounds unintentionally in the past year), exhaustion, slow walking speed, weakness (based on grip strength), and low physical activity (measured in Kcal) to define frailty. However, we do not have data on unintentional weight loss in the past year, grip strength and physical activity measured in Kcal in the REGARDS study. Using only frailty indicators included in the definition of Fried et al. [2] which are available in the REGARDS study (i.e., exhaustion and slow walk) would have limited our ability to identify participants who are frail in our study. Therefore, we decided to include low BMI (as a proxy for weight loss), weakness based on chair stand (as a proxy for weakness), and history of falls (as another proxy for weakness) as frailty indicators. We then included sensitivity analyses to determine the robustness of our definition. As a rationale for including history of falls in our definition, we note that many prior studies have shown that frail adults are more likely to fall versus their counterparts who are not frail [19, 20]. We defined being frail as having ≥ 3 of the 5 indicators, intermediate-frailty as having 1–2 indicators, and not frail as having 0 indicators.

### Gaps in care coordination

We assessed seven gaps in care coordination based on responses to eight questions from a survey on experiences with healthcare, as previously described by Kern et al. [16]. Examples of such questions include: “In the last 6 months, did you get the help you needed from your personal doctor’s office to manage your care among different providers and services?” (Never, Sometimes, Usually, or Always), “In general, do you think the doctors you see communicate with each other about your care?” (Yes, No, or I Don’t Know) and “In general, how would you describe the coordination among all of the different health professionals that you see?” (Excellent, Very Good, Good, Fair, or Poor). The seven gaps in care coordination, and the corresponding questions and definitions are shown in Supplemental Table 2.

### Preventable adverse events

We used a questionnaire previously tested by Kern et al. [16] to assess 4 outcomes that occurred in the past 12 months and participants thought would have been preventable through better care coordination: (1) A problem because different doctors prescribed medications which may not go well when taken together, (2) The need to repeat a test (e.g., blood test, x-ray) because the initial results were unavailable, (3) An emergency department visit that participants thought would have been prevented through better care coordination, and (4) A hospitalization that participants thought would have been prevented through better care coordination. The questions to assess the 4 preventable adverse events are shown in Supplemental Table 3.

### Covariates

We used data collected at the second REGARDS study CATI and in-home examination to assess potential confounders selected a priori, including age, income, marital status, hypertension, dyslipidemia, diabetes, prior coronary revascularization, chronic kidney disease (CKD), atrial fibrillation, self-rated health, disability with activities of daily living (ADL), and instrumental ADL (IADL), and social support. We used data collected at the REGARDS study baseline to assess other potential confounders selected a priori including gender, race, education, geographic region of residence, and rural residence as information on these variables was not collected at the second REGARDS study CATI and in-home examination. History of stroke and myocardial infarction were defined using data collected at the REGARDS study baseline and during the second REGARDS study CATI and in-home examination supplemented by adjudicated events identified during follow-up. Supplemental Table 4 and Supplemental Fig. 2 provides further detail on how variables listed above were assessed.

### Statistical analysis

We calculated descriptive statistics of participants characteristics, overall and among those who were frail, intermediate-frail and not frail, separately. We used Analysis of Variance, Wilcoxon rank sum tests and chi-square tests, as appropriate, to compare participant characteristics by frailty status.

We calculated the prevalence of having any gap in care coordination, overall and by frailty status. We used Poisson regression models with robust variance estimators to calculate prevalence ratios (PRs) and 95% confidence intervals (CIs) for having any gap in care coordination among participants who were frail and intermediate-frail versus those not frail, and to test for trend across frailty status categories [21, 22]. Model 1 was unadjusted. Model 2 included adjustment for age, gender, race, education, annual household income, marital status, geographic region of residence, and rural residence. Model 3 included adjustment for variables in model 2 and hypertension, diabetes, dyslipidemia, history of myocardial infarction, coronary revascularization, or stroke, CKD, atrial fibrillation and self-rated health. We repeated model 3 using multiple imputation with chained equations to account for missing data on covariates (Supplemental Table 5) [23]. We consider the model using multiple imputation as our final model.

We estimated the number of questions to which participants reported gaps in care coordination, ranging from 0 to 7, by frailty status. The count of questions showed overdispersion with excess zeros. Therefore, we used marginalized zero-inflated Poisson regression models with adjustment for covariates as described above to calculate the ratio and 95% CIs for the mean number of questions to which participants reported gaps in care coordination among participants who were frail and intermediate-frail versus those not frail [24].

We analyzed the prevalence of any preventable adverse event by frailty status using the same approach as described above for the prevalence of any gap in care coordination. We could not estimate the association between frailty and number of preventable adverse events as few participants had 2, 3, or 4 adverse events. In a separate analysis, we determined the association between having a gap in care coordination (or not) and the prevalence of any preventable adverse event among participants who were intermediate-frail and frail, combined and separately, using the same approach described above for the analysis of the association of frailty with any gap in care coordination.

We conducted four sensitivity analyses. First, we excluded weakness from the frailty definition because we defined weakness using a chair stand test whereas Fried et al., used grip strength [2]. Second, we excluded history of falls from the frailty definition because history of falls was not included in the frailty index by Fried et al. [2]. In both sensitivity analyses described above, frailty was defined as having 3–4 indicators, intermediate-frailty as having 1–2 indicators and not frail as having 0 indicators. Third, we repeated the main analyses including adjustment for ADL and IADL in Models 2 and 3 described above as these covariates may be markers of frailty. Data on social support in REGARDS were collected through a self-administered questionnaire that participants had to complete and return by mail. We did not conduct multiple imputation on social support for the main analysis given the large proportion of missing data (25.7%) and because it is unclear whether data were missing at random. In the fourth sensitivity analysis, we repeated the main analyses restricted to participants with valid data on social support (*n* = 3,733) and included adjustment for this variable in Models 2 and 3 described above. All analyses were conducted using SAS, version 9.4 (SAS Institute, Inc, Cary, NC).

## Results

### Participants’ characteristics

Overall, 2,398 (47.7%) participants were not frail, 2,436 (48.5%) were intermediate-frail, and 190 (3.8%) were frail. Participants who were intermediate-frail or frail were older versus those not frail (Table 1). Participants who were frail had more medical conditions, were more likely to self-rate their health as fair or poor, and were more likely to report disability in ≥ 1 ADL or IADL versus those intermediate-frail or not frail.

**Table 1:** Characteristics of REGARDS study participants included in the current analysis

Characteristics	Overall	Not frail	Intermediate-frail	Frail	*P*-value
	(*n* = 5024)	(*n* = 2398)	(*n* = 2436)	(*n* = 190)	
Demographic characteristics					
Age, years	73.5 ± 6.1	72.9 ± 5.8	74.0 ± 6.2	74.2 ± 6.8	< 0.001
Female	2799 (55.7)	1269 (52.9)	1402 (57.6)	128 (67.4)	< 0.001
Black	1615 (32.2)	763 (31.8)	784 (32.2)	68 (35.8)	0.528
Annual income < $25,000	937 (19.8)	359 (15.8)	512 (22.4)	66 (38.6)	< 0.001
Less than high school education	254 (5.1)	94 (3.9)	137 (5.6)	23 (12.1)	< 0.001
Marital status, Married	2972 (59.3)	1510 (63.2)	1378 (56.7)	84 (44.2)	< 0.001
Geographic region of residence^a^					
Stroke belt	1602 (31.9)	745 (31.1)	799 (32.8)	58 (30.5)	0.579
Stroke buckle	1079 (21.5)	535 (22.3)	503 (20.7)	41 (21.6)	
Other US regions	2343 (46.6)	1118 (46.6)	1134 (46.6)	91 (47.9)	
Rural area^b^	499 (11.0)	229 (10.6)	252 (11.4)	18 (9.9)	0.604
Clinical characteristics					
Hypertension	3461 (68.9)	1591 (66.4)	1718 (70.5)	152 (80.0)	< 0.001
Hyperlipidemia	3102 (63.8)	1463 (62.7)	1508 (64.1)	131 (72.0)	0.039
Diabetes	1240 (25.5)	525 (22.5)	643 (27.4)	72 (39.1)	< 0.001
History of myocardial infarction	746 (15.4)	291 (12.5)	414 (17.6)	41 (22.8)	< 0.001
Prior coronary revascularization	796 (15.9)	333 (13.9)	425 (17.5)	38 (20.0)	0.001
History of stroke	285 (5.7)	97 (4.1)	165 (6.8)	23 (12.2)	< 0.001
Chronic kidney disease	1885 (40.3)	828 (36.5)	965 (43.1)	92 (52.9)	< 0.001
Atrial fibrillation	581 (12.1)	237 (10.3)	315 (13.6)	29 (16.2)	0.001
Self-rated health					
Excellent	652 (13.0)	412 (17.3)	232 (9.6)	8 (4.2)	< 0.001
Very good/good	3684 (73.6)	1807 (75.6)	1773 (73.1)	104 (54.7)	
Fair/poor	669 (13.4)	170 (7.1)	421 (17.4)	78 (41.1)	
Self-reported disability					
Disability in ≥ 1 ADL task	556 (11.1)	113 (4.7)	371 (15.3)	72 (37.9)	< 0.001
Disability in ≥ 1 IADL task	1596 (31.8)	489 (20.4)	976 (40.1)	131 (69.0)	< 0.001
Frailty indicators					
Low BMI	40 (0.8)	0 (0.0)	32 (1.3)	8 (4.2)	< 0.001
Exhaustion	611 (12.3)	0 (0.0)	479 (19.8)	132 (69.5)	< 0.001
Slow walk	1012 (20.5)	0 (0.0)	864 (36.1)	148 (77.9)	< 0.001
Weakness	920 (20.1)	0 (0.0)	784 (36.2)	136 (85.5)	< 0.001
History of falls	1156 (23.1)	0 (0.0)	990 (40.8)	166 (87.8)	< 0.001
Ambulatory utilization					
Number of ambulatory visits in the past 12 months, median (25^th^, 75^th^ percentiles)	5 (3,8)	5 (3,7)	5 (3,8)	6 (4,10)	< 0.001
Number of ambulatory providers in the past 12 months, median (25^th^, 75^th^ percentiles)	3 (2,4)	3 (2,4)	3 (2,4)	3 (3,5)	< 0.001

Values are mean ± standard deviation or frequencies (%)

*ADL* Activities of daily living, *IADL* Instrumental activities of daily living, *BMI* body mass index

^a^ Stroke buckle includes coastal North Carolina, South Carolina and Georgia. Stroke belt includes the remaining parts of North Carolina, South Carolina and Georgia, and Tennessee, Mississippi, Alabama, Louisiana and Arkansas. Other US regions includes the remaining 40 contiguous US states and the District of Columbia

^b^ Rural area was defined based on census tract data

Definitions for frailty indicators are provided in Supplemental Table 1. Definitions for other participant characteristics presented in this Table are provided in Supplemental Table 4

### Frailty and gaps in care coordination

The prevalence of having any gap in care coordination was 37.0%, 40.8%, and 51.1% among participants who were not frail, intermediate-frail, and frail, respectively. The frequency of each gap in care coordination, overall and by frailty status, is presented in Supplemental Table 6. In the final model, participants who were intermediate-frail (PR, 1.09 [95% CI, 1.02 – 1.18]) and frail (PR, 1.34 [95% CI, 1.15 – 1.56]) had a higher prevalence of any gap in care coordination compared to their counterparts who were not frail (Table 2). Participants intermediate-frail and frail were also more likely to report gaps in care coordination in a higher number of questions versus those who were not frail (Supplemental Table 7).

**Table 2:** Association between frailty and any gap in care coordination

	**Not frail**	**Intermediate-frail**	**Frail**	**P-trend**
N	2398	2436	190	
N (%) with ≥ 1 gap in care coordination	888 (37.0)	994 (40.8)	97 (51.1)	< 0.001
Prevalence ratios (95% confidence intervals)				
Model 1 (*n* = 5,024)	1 (Ref)	1.10 (1.03 – 1.18)	1.38 (1.19 – 1.60)	< 0.001
Model 2 (*n* = 4,289)	1 (Ref)	1.07 (0.99 – 1.16)	1.31 (1.12 – 1.55)	0.004
Model 3 (*n* = 3,713)	1 (Ref)	1.10 (1.01 – 1.20)	1.41 (1.18 – 1.68)	0.001
Final model^a^ (*n* = 5,024)	1 (Ref)	1.09 (1.02 – 1.18)	1.34 (1.15 – 1.56)	< 0.001

Model 1 is unadjusted

Model 2 includes adjustment for age, gender, race, education, annual household income, marital status, geographic region of residence, and rural area

Model 3 includes adjustment for the variables in model 2 and hypertension, hyperlipidemia, diabetes, history of myocardial infarction, prior coronary revascularization, history of stroke, chronic kidney disease, atrial fibrillation, and self-rated health

^a^ In the final model, multiple imputation was used to retain participants with missing data in covariates

The higher prevalence of any gap in care coordination among participants who were frail versus not frail persisted in sensitivity analyses excluding weakness from the frailty definition, and adjusting for ADL/IADL, separately (Supplemental Table 8, panel A and C, respectively). There was also a trend for a higher prevalence of any gap in care coordination among intermediate-frail and frail participants versus those not frail in the sensitivity analyses excluding history of falls from the frailty definition (p-trend in the final model < 0.001, Supplemental Table 8**, **panel B) and adjusting for social support (p-trend in the final model 0.043, Supplemental Table 8**, **panel D).

### Frailty and preventable adverse events

The prevalence of preventable adverse events was 7.0%, 11.3%, 20.0% among participants who were not frail, intermediate-frail and frail, respectively. The frequency of each preventable adverse event by frailty status is presented in Supplemental Table 9. In the final model, the PR for preventable adverse events among participants who were intermediate-frail and frail versus not frail was 1.47 (95% CI, 1.22 – 1.77) and 2.24 (95% CI, 1.60 – 3.14), respectively (Table 3). The higher prevalence of preventable adverse events among participants who were frail versus not frail was also present in separate sensitivity analyses removing weakness and history of falls from the frailty definition, separately, adjusting for ADL/IADL, and adjusting for social support (Supplemental Table 10).

**Table 3:** Association between frailty and any preventable adverse event

	**Not frail**	**Intermediate-frail**	**Frail**	**P-trend**
N	2398	2436	190	
N (%) with ≥ 1 preventable adverse event	167 (7.0)	276 (11.3)	38 (20.0)	< 0.001
Prevalence ratios (95% confidence intervals)				
Model 1 (*n* = 5,024)	1 (Ref)	1.63 (1.35 – 1.96)	2.87 (2.09 – 3.95)	< 0.001
Model 2 (*n* = 4,289)	1 (Ref)	1.66 (1.36 – 2.04)	2.72 (1.89 – 3.92)	< 0.001
Model 3 (*n* = 3,713)	1 (Ref)	1.60 (1.28 – 2.01)	2.47 (1.64 – 3.73)	< 0.001
Final model^a^ (*n* = 5,024)	1 (Ref)	1.47 (1.22 – 1.77)	2.24 (1.60 – 3.14)	< 0.001

Model 1 is unadjusted

Model 2 includes adjustment for age, gender, race, education, annual household income, marital status, geographic region of residence and rural area

Model 3 includes adjustment for the variables in model 2 and hypertension, hyperlipidemia, diabetes, history of myocardial infarction, prior coronary revascularization, history of stroke, chronic kidney disease, atrial fibrillation, and self-rated health

^a^ In the final model, multiple imputation was used to retain participants with missing data in covariates

### Gaps in care coordination and preventable adverse events in participants with intermediate-frailty or frailty

Among participants who were intermediate-frail or frail, the prevalence of any preventable adverse event was higher among those with any versus none gap in care coordination (14.7% vs. 10.0%, respectively, Table 4). In the final model, having any versus none gap in care coordination was associated with a higher prevalence of having any preventable adverse event (PR, 1.45 [95% CI, 1.18 – 1.78]). The higher prevalence of any preventable adverse event associated with having any versus none gap in care coordination was present in participants who were frail and intermediate-frail, separately, although the association was not statistically significant among those who were frail. The higher prevalence of preventable adverse events associated with having any gap in care coordination among participants who were intermediate-frail or frail was also present in sensitivity analyses excluding weakness and history of falls from the frailty definition, separately, adjusting for ADL/IADL, and adjusting for social support (Supplemental Table 11).

**Table 4:** Association between any gap in care coordination and preventable adverse events, among participants with intermediate-frailty or frailty, and separately by intermediate-frailty and frailty

	No gap in care coordination	Gap in care coordination
**Participants with intermediate-frailty or frailty**		
N	1535	1091
Participants with ≥ 1 preventable adverse event, n (%)	154 (10.0)	160 (14.7)
Prevalence ratios (95% confidence intervals) for ≥ 1 preventable adverse event		
Model 1 (*n* = 2,626)	1 (Ref)	1.46 (1.19 – 1.80)
Model 2 (*n* = 2,234)	1 (Ref)	1.34 (1.07 – 1.68)
Model 3 (*n* = 1,892)	1 (Ref)	1.38 (1.08 – 1.77)
Final model^a^ (*n* = 2,626)	1 (Ref)	1.45 (1.18 – 1.78)
**Participants with intermediate-frailty**		
N	1442	994
Participants with ≥ 1 preventable adverse event, n (%)	139 (9.6)	137 (13.8)
Prevalence ratios (95% confidence intervals) for any preventable adverse event		
Model 1 (*n* = 2,436)	1 (Ref)	1.43 (1.15 – 1.78)
Model 2 (*n* = 2,070)	1 (Ref)	1.34 (1.05 – 1.70)
Model 3 (*n* = 1,756)	1 (Ref)	1.33 (1.02 – 1.74)
Final model^a^ (*n* = 2,436)	1 (Ref)	1.44 (1.15 – 1.79)
**Participants with frailty**		
N	93	97
Participants with ≥ 1 preventable adverse event, n (%)	15 (16.1)	23 (23.7)
Prevalence ratios (95% confidence intervals) for any preventable adverse event		
Model 1 (*n* = 190)	1 (Ref)	1.47 (0.82 – 2.64)
Model 2 (*n* = 164)	1 (Ref)	1.11 (0.57 – 2.18)
Model 3 (*n* = 136)	1 (Ref)	1.51 (0.68 – 3.38)
Final model^a^ (*n* = 190)	1 (Ref)	1.29 (0.69 – 2.42)

Model 1 is unadjusted

Model 2 includes adjustment for age, gender, race, education, annual household income, marital status, geographic region of residence and rural area

Model 3 includes adjustment for the variables in model 2 and hypertension, hyperlipidemia, diabetes, history of myocardial infarction, prior coronary revascularization, history of stroke, chronic kidney disease, atrial fibrillation, and self-rated health

^a^ In the final model, multiple imputation was used to retain participants with missing data in covariates

## Discussion

In this national cohort of older community-dwelling adults, frail participants frequently reported gaps in care coordination (51.1%) and preventable adverse events (20.0%). Participants who were intermediate-frail and frail had a 9% and 34% greater adjusted prevalence, respectively, of experiencing any gap in care coordination compared to those who were not frail. Also, participants who were intermediate-frail and frail had a 47% and 124% greater adjusted prevalence respectively, of experiencing any preventable adverse event compared to those who were not frail. Intermediate-frail or frail older adults who experienced a gap in care coordination had a 45% greater adjusted prevalence of experiencing any preventable adverse event than those who did not experience a gap in care coordination.

Patients have a distinct vantage point and may be able to identify gaps in their care coordination before providers do [25]. Patients may also be the most reliable reporters of some aspects of the health care process [26]. A prior study using the patient Open Notes reporting system showed that 64% of the safety concerns reported by patients were validated upon clinician review and that 57% of confirmed problems resulted in a change in patient care [27]. Therefore, patients’ perspectives could be used to inform interventions to improve quality of care. To our knowledge, no other study has measured frail older adults’ perceptions of gaps in care coordination and preventable adverse events. In a previously published survey, 35% of US adults 65 years and older reported having experienced a gap in healthcare coordination [28]. Recently, using data from the REGARDS study, Kern et al., reported that 38% of older adults in the US perceive having a gap in their care coordination [16]. However, neither of those studies focused on frail older adults. Results from the current study expand on prior knowledge, suggesting that the likelihood of a gap in care coordination is higher among intermediate-frail and frail older adults compared to those who are not frail.

Although prior studies have assessed the association between frailty and adverse events, including hospitalizations [2, 29] and emergency department visits [6, 29], these studies did not account for whether these events were perceived by patients to be potentially preventable. Also, some of these studies were conducted in a single state [6, 29]. For example, the study by McNallan et al. [6], using health records from older adults with heart failure from 3 counties in Minnesota, showed that those who were intermediate-frail and frail had a higher risk for hospitalizations and emergency department visits versus their counterparts without frailty. In another study conducted among older adults from the Boston area of Massachusetts, Kiely et al. [29, reported that those who were intermediate-frail or frail had a higher odds for having a self-reported hospitalization and emergency department visit. However, authors did not specify if participants perceived these events as potentially preventable through better care coordination.

Among intermediate-frail and frail older adults included in the current study, having any versus none gap in care coordination was associated with a higher likelihood for a self-reported adverse event. Some studies have assessed the association between patient perceived coordination of care and increased odds of adverse events in the general population [26, 30, 31]. However, these studies did not conduct subgroup analyses among frail older adults, and analyzed a limited number of potential adverse events that may be associated with care coordination gaps. We expanded the list of adverse events that have previously been considered to include laboratory-related adverse events, medication-related adverse events, as well as preventable emergency department visits and hospitalizations. Therefore, identifying patients reporting gaps in care, may be useful to direct interventions to prevent adverse events to those with higher risk, something which needs to be confirmed in future studies.

The strengths of the current analysis include the national sampling frame, large sample size, use of many previously validated survey questions, and adjustment for clinically detailed potential confounders. However, the results of the current study should be interpreted in the context of some potential limitations. This was a cross-sectional study, thus, causality cannot be determined. The prevalence of frailty in our study (3.8%) was lower than the prevalence found in other studies (ranging from 4.0% to 34.8%) [2, 32–34]. The wide range of estimates reported across studies may be explained by differences in the study population and the frailty definition. In the current analysis, in addition to using an adapted version of the definition of frailty by Fried et al. 2, we included participants who have been followed for over 10 years. Therefore, participants included in our analysis may be healthier and less likely to be frail than the general population of US adults ≥ 65 years of age. Participants may not accurately recall their experiences with healthcare. We excluded participants with cognitive impairment from the current analysis as this may lead to differential recall by frailty status. Five of the survey questions about gaps in care coordination referred to events in the last 6 months before the survey, as 6 months is standard for measuring recall related to satisfaction with healthcare [35]. The questionnaire to identify adverse events was based on self-report which may not be accurate. However, events reported by participants may still represent an opportunity to improve quality of care. Preventable adverse events were reported for the last 12 months, as some of them were relatively unfrequent [36]. Thus, it is possible that some preventable adverse events preceded the gaps in care coordination reported by participants. Finally, we cannot determine from the data in this study why older adults who are frail were more likely to report gaps in care coordination and preventable adverse events versus those not frail.

## Conclusion

In this national cohort, frail and intermediate-frail older adults were more likely to report a gap in care coordination compared to those not frail. Frail and intermediate-frail older adults were also more likely than non-frail older adults to report the occurrence of an adverse event that they thought could have been preventable through better care coordination, including a repeated test, drug-drug interaction, emergency department visit, or hospitalization. Among frail and intermediate-frail older adults, reporting a gap in care coordination was associated with a higher likelihood of reporting a preventable adverse event. Targeted strategies to address patient-reported concerns regarding care coordination among intermediate-frail and frail older adults may be warranted.

## Supplementary Information

**Additional file 1: Supplemental Table 1**. Indicators of frailty used in the current analysis and the corresponding component of the phenotype described by Fried et al. [2]. **Supplemental Table 2**. Definition of the gaps in care coordination assessed in the survey module on experiences with healthcare [16]. **Supplemental Table 3**. Definitions of the preventable adverse events assessed in the survey module on experiences with healthcare [16]. **Supplemental Table 4**. Definitions of covariates used in this analysis. **Supplemental Table 5**. Number and percentage of participants with missing data for each variable in the main analysis (*n*=5,024). **Supplemental Table 6**. Frequency of self-reported gaps in care coordination overall and by frailty status. **Supplemental Table 7**. Association between frailty and the number of questions to which participants reported gaps in care coordination. **Supplemental Table 8**. Sensitivity analyses for the association between frailty and any gap in care coordination. **Supplemental Table 9**. Frequency of self-reported preventable adverse events in the previous 12 months by frailty status. **Supplemental Table 10**. Sensitivity analyses for the association between frailty and any preventable adverse event. **Supplemental Table 11**. Sensitivity analyses for the association between any gap in care coordination and any preventable adverse events, among participants with intermediate-frailty or frailty. **Supplemental Figure 1**. Flow-chart of REGARDS study participants included in the current analysis. **Supplemental Figure 2**. Timing of data collection for variables used in this analysis.

## Data Availability

The REGARDS data are available to qualified researchers trained in human subject confidentiality research upon reasonable request to the REGARDS executive committee at http://regardsstudy.org. Other information about the current analysis is available from the corresponding author.

## Abbreviations

US: United States
REGARDS: REasons for Geographic And Racial Differences in Stroke
SIS: Six Item Screener
BMI: Body mass index
CKD: Chronic kidney disease
ADL: Activities of daily living
IADL: Instrumental activities of daily living
RRs: Risk ratios
CIs: Confidence intervals
