# Supplementary material for: Frailty, gaps in care coordination, and preventable adverse events
Source: BMC Geriatr. 2022 Jun 2;22:476. doi: 10.1186/s12877-022-03164-7 (PMC9164877; doi:10.1186/s12877-022-03164-7)
Supplement: Supplementary file 1 — Additional file 1: Supplemental Table 1. Indicators of frailty used in the current analysis and the corresponding component of the phenotype described by Fried et al. [2]. Supplemental Table 2. Definition of the gaps in care coordination assessed in the survey module on experiences with healthcare [16]. Supplemental Table 3. Definitions of the preventable adverse events assessed in the survey module on experiences with healthcare [16]. Supplemental Table 4. Definitions of covariates used in this analysis. Supplemental Table 5. Number and percentage of participants with missing data for each variable in the main analysis (n=5,024). Supplemental Table 6. Frequency of self-reported gaps in care coordination overall and by frailty status. Supplemental Table 7. Association between frailty and the number of questions to which participants reported gaps in care coordination. Supplemental Table 8. Sensitivity analyses for the association between frailty and any gap in care coordination. Supplemental Table 9. Frequency of self-reported preventable adverse events in the previous 12 months by frailty status. Supplemental Table 10. Sensitivity analyses for the association between frailty and any preventable adverse event. Supplemental Table 11. Sensitivity analyses for the association between any gap in care coordination and any preventable adverse events, among participants with intermediate-frailty or frailty. Supplemental Figure 1. Flow-chart of REGARDS study participants included in the current analysis. Supplemental Figure 2. Timing of data collection for variables used in this analysis. [file 12877_2022_3164_MOESM1_ESM.docx]

**Supplemental Materials**

**Frailty, self-reported gaps in care coordination, and preventable adverse events**

Oluwasegun P. Akinyelure, MD, MPH^1^, Calvin C. Colvin, MSPH^1^, Madeline R. Sterling, MD, MPH^2^, Monika M. Safford, MD^2^, Paul Muntner, PhD^1^, Lisandro D. Colantonio, MD, PhD^1^, Lisa M. Kern, MD, MPH^2^

^1^Department of Epidemiology, University of Alabama at Birmingham, Birmingham, AL, United States.

^2^Department of Medicine, Weill Cornell Medicine, New York, NY, United States.

**Corresponding Author:**

Lisa M. Kern, MD, MPH.

Department of Medicine

Weill Cornell Medicine

420 East 70^th^ Street, Box 331

New York, NY 10021, USA

Phone: (646) 962-5889

Email: [lmk2003@med.cornell.edu](mailto:lmk2003@med.cornell.edu)

**Supplemental Table 1.** Indicators of frailty used in the current analysis and the corresponding component of the phenotype described by Fried et al.^1^

| **Indicator of frailty** | **Definition (using data collected during the second REGARDS study examination)** | **Corresponding components of the phenotype described by Fried et al.*** |
| --- | --- | --- |
| Low BMI | BMI < 18.5 kg/m^2^ | Unintentional weight loss |
| Exhaustion | Replying ≥ 3 days to either of the following 2 questions from the CESD scale:^2^   - How many times during the past week did you feel that everything you did was an effort? - How many times during the past week did you feel you could not get going? | Exhaustion, defined by replying ≥ 3 days to either of the following 2 questions from the CESD scale:^2^   - How many times during the past week did you feel that everything you did was an effort? - How many times during the past week did you feel you could not get going? |
| Slow walk | Time to walk 8 feet at usual pace ≥ 80^th^ percentile of the distribution stratified by gender and height. Two trials were performed. Consistent with a prior study,^3^ the shortest time (i.e., the fastest trial) was used for analysis. The time scores were stratified by gender and then by median height (2 strata per gender). In each stratum, those in the highest quintile (i.e., ≥ 80^th^ percentile) were considered to have slow walk. | Slow walk, defined as a time to walk 15 feet at usual pace ≥ 80^th^ percentile of the distribution stratified by gender and height. |
| Weakness | Time to complete 5 chair stands ≥ 80^th^ percentile of the distribution stratified by gender and body mass index. The distribution was stratified by gender and then by body mass index in quartiles (4 strata per gender). In each stratum, those in the highest quintile (i.e., ≥ 80^th^ percentile) were considered to be weak. | Weakness was defined by a maximal grip strength in the dominant hand using a Jamar hand-held dynamometer <20^th^ percentile of the distribution stratified by gender and body mass index. |
| History of fall | Self-reported history of fall. | N/A |

REGARDS: REasons for Geographic And Racial Differences in Stroke.

BMI: body mass index.

CESD: Center for Epidemiologic Studies Depression Scale.^2^

* The remaining component of the frailty phenotype described by Fried et al. (i.e., low physical activity) was not analyzed as these data are unavailable in REGARDS.

**Supplemental Table 2**. Definition of the gaps in care coordination assessed in the survey module on experiences with healthcare.^4^

| **Questions** | **Definition of gaps** |
| --- | --- |
| 1. In the past 6 months, when you visited your personal doctor for a scheduled appointment, how often did he or she have your medical records or other information about your care? | Responding “sometimes” or “never” was considered a gap in care coordination |
| 1. In the past 6 months, how often did you and your personal doctor talk about all the prescription medicines you were taking? | Responding “sometimes” or “never” was considered a gap in care coordination |
| 1. In the past 6 months, how often did your personal doctor seem informed and up-to-date about the care you got from specialists? | Responding “sometimes” or “never” was considered a gap in care coordination |
| 1. In the past 6 months, did you get the help you needed from your personal doctor’s office to manage your care among these different providers and services? | Responding “somewhat” was considered a gap in care coordination |
| 1. In the past 6 months, when your personal doctor ordered a blood test, x-ray, or other test for you, how often did someone from your personal doctor’s office follow up to give you those results?* 2. In the past 6 months, when your personal doctor ordered a blood test, x-ray, or other test for you, how often did you get those results as soon as you needed them?* | Responding “sometimes” or “never” to either of these two questions was considered a gap in care coordination |
| 1. In general, do you think the doctors that you see communicate with each other about your care? | Responding “no” was considered a gap in care coordination |
| 1. In general, how would you describe the coordination among all of the different health professionals that you see? | Responding “fair” or “poor” was considered a gap in care coordination |

*These two questions were combined as per the Consumer Assessment of Healthcare Providers and Systems (CAHPS) scoring rule.^5^

**Supplemental Table 3**. Definitions of the preventable adverse events assessed in the survey module on experiences with healthcare.^4^

| **Events** | **Questions** | **Definition of adverse events** |
| --- | --- | --- |
| Drug-drug interaction | In the past 12 months, have you had a problem with your medications, because different doctors prescribed medications that did not go well together? | Participants responding “Yes” were considered to have had a self-reported drug-drug interaction. |
| Repeat Testing | In the past 12 months, have you been told to repeat a blood test, x-ray, or other test that you had had in the past two weeks, because your doctor did not have the results of the first test? | Participants responding “Yes” were considered to have had a self-reported repeat test. |
| Emergency department visit | 1. In the past 12 months, have you had one or more emergency department visits?      1. Do you think one or more of your emergency department visits could have been prevented? 2. Do you think one or more of your emergency department visits could have been prevented by better communication across your different doctors and other healthcare providers? | Participants responding “Yes” to all three questions were considered to have had a self-reported preventable emergency department visit preventable with better communication. |
| Hospital admission | 1. In the past 12 months, have you been admitted to the hospital at least once? 2. Do you think one or more of your hospitalizations could have been prevented? 3. Do you think one or more of your hospitalizations could have been prevented by better communication across your different doctors and other healthcare providers? | Participants responding “Yes” to all three questions were considered to have had a self-reported hospital admission preventable with better communication. |

**Supplemental Table 4.** Definitions of covariates used in this analysis.

| Covariate | Definition |
| --- | --- |
| Age | Self-reported during a computer assisted telephone interview conducted in conjunction with the REGARDS second study examination (2013–2016). |
| Gender | Self-reported as Male or Female at the REGARDS baseline examination (2003–2007). |
| Race | Self-reported as Non-Hispanic Black or White at the REGARDS baseline examination (2003–2007). |
| Annual income | Annual household income from all sources was self-reported at the REGARDS second study examination (2013–2016) and categorized as < $25,000 or ≥ $25,000 for the current analysis. |
| Education | Self-reported at the REGARDS baseline examination (2003–2007) and categorized as less than or greater than high school education for the current analysis.  Less than high school education was defined as less than high school graduate or no General Educational Development (GED) certificate. |
| Marital status | Self-reported at the REGARDS second study examination (2013–2016) and categorized as married or not married for the current analysis. |
| Region of residence | Based on home addresses self-reported at the REGARDS baseline examination (2003–2007). Region of residence was categorized into stroke buckle (coastal North Carolina, South Carolina and Georgia), stroke belt (the remaining parts of North Carolina, South Carolina and Georgia, and Tennessee, Mississippi, Alabama, Louisiana and Arkansas), and other US regions (the remaining 40 contiguous US states and the District of Columbia). |
| Rural residence | Based on home addresses self-reported at the REGARDS baseline examination (2003–2007). Rural area was defined based on census tract data. |
| Hypertension | Defined as systolic blood pressure ≥ 140 mm Hg or diastolic blood pressure ≥ 90 mm Hg or self-reported use of antihypertensive medication among participants with diagnosed hypertension during the REGARDS second study examination (2013–2016).  Blood pressure was measured during the REGARDS second study examination using an aneroid sphygmomanometer following an standardized protocol. |
| Dyslipidemia | Defined as total cholesterol ≥ 240 mg/dL, low-density lipoprotein cholesterol ≥ 160 mg/dL, high-density lipoprotein cholesterol ≤ 40 mg/dL, or self-reported lipid-modifying medication use at the REGARDS second study examination (2013–2016). |
| Diabetes | Defined as fasting glucose ≥ 126 mg/dL, non-fasting glucose ≥ 200 mg/dL, or self-reported glucose-lowering medication use at the REGARDS second study examination (2013–2016). |
| Myocardial infarction | Defined as evidence of myocardial infarction on a study electrocardiogram conducted during the REGARDS second study examination (2013–2016) or self-reported history of myocardial infarction at the second study examination supplemented with adjudicated myocardial infarction events between baseline and the second study examination.^4,6^ |
| Prior coronary revascularization | Defined as self-reported coronary angioplasty, stenting, or bypass surgery at the REGARDS second study examination (2013–2016). |
| Stroke | Defined as self-reported history of stroke events at the REGARDS baseline examination (2003–2007), or an adjudicated stroke event between baseline and the second study examination.^4,7^ |
| Chronic kidney disease | Defined as urinary albumin-to-creatinine ratio ≥ 30 mg/g or estimated glomerular filtration rate (eGFR) < 60 mL/min/1.73m^2^. eGFR was calculated using serum creatinine from blood sample collected at the REGARDS second study examination (2013–2016), and the Chronic Kidney Disease (CKD) Epidemiology Collaboration equation. Urinary albumin and creatinine were measured from a spot urine sample using a nephelometer (BNII ProSpec nephelometer), and the rate-blanked Jaffe assay (the Modular-P chemistry analyzer), respectively. |
| Atrial fibrillation | Defined as evidence of atrial fibrillation on the study electrocardiogram conducted during the REGARDS second study examination or self-reported diagnosis of atrial fibrillation at the REGARDS second study examination (2013–2016). |
| Self-rated health | Categorized as Excellent, Very good/Good, Fair/Poor. Self-rated heath was measured using the 1^st^ question in the Short Form 12 survey collected at the REGARDS second study examination (2013–2016. |
| Activities of daily living | Measured during the second study examination. Disability in ≥ 1 activities of daily living task was defined by self-reported difficulty or needing assistance performing any of the five activities of daily living tasks which include getting out of bed or chair, eating including feeding oneself, dressing oneself, bathing oneself, using the toilet including getting to the toilet. |
| Instrumental activities of daily living | Measured during the second study examination. Disability in ≥ 1 instrumental activities of daily living task was defined by self-reported difficulty or needing assistance performing any of the seven instrumental activity of daily living tasks which includes doing household chores, purchasing items at the store, planning and preparing meals, managing money such as paying bills, using a telephone or cellphone, taking medications on time and as prescribed by the doctor, traveling by vehicles to places beyond walking distance. |
| Social support | Measured during the second study examination with a self-administered questionnaire. Social support was assessed using 6 questions: (1) Is there someone available to you whom you can count on to listen to you when you need to talk? (2) Is there someone available to you to give you good advice about a problem? (3) Is there someone available to you who shows you love and affection? (4) Is there someone available to you to help you with daily chores? (5) Can you count on anyone to provide you with emotional support (talking over problems or helping make difficult decisions)? (6) Do you have as much contact as you would like with someone you feel close to, someone in whom you can trust and confide? Participants’ responses for each question were: None of the time, A little of the time, Some of the time, Most of the time, All of the time. Each question was scored from 0 to 4 where “0” is “None of the time” and “4” is “All of the time”. Sum of the scores for the six questions assessing social support. Range from 0 to 24 with higher values representing more social support. |

REGARDS: REasons for Geographic And Racial Differences in Stroke.

**Supplemental Table 5.** Number and percentage of participants with missing data for each variable in the main analysis (n=5,024).

| Participant characteristics | Participants with missing data  N (%) |
| --- | --- |
| Socio-demographic characteristics |  |
| Age | 0 (0.0) |
| Gender | 0 (0.0) |
| Race | 0 (0.0) |
| Annual income <$25,000 | 289 (5.8) |
| Less than high school education | 0 (0.0) |
| Marital status | 12 (0.2) |
| Geographic region of residence | 0 (0.0) |
| Rural residence | 477 (9.5) |
| Clinical characteristics |  |
| Hypertension | 1 (0.0) |
| Hyperlipidemia | 158 (3.1) |
| Diabetes | 159 (3.2) |
| Myocardial infarction | 165 (3.3) |
| Prior coronary revascularization | 18 (0.4) |
| Stroke | 13 (0.3) |
| Chronic kidney disease | 342 (6.8) |
| Atrial fibrillation | 210 (4.2) |
| Self-rated health | 19 (0.4) |
| Disability |  |
| ≥ 1 ADL tasks | 8 (0.2) |
| ≥ 1 IADL tasks | 8 (0.2) |
| Social support | 1291 (25.7) |
| Ambulatory utilization |  |
| Number of ambulatory visits in the past 12 months | 0 (0.0) |
| Number of ambulatory providers in the past 12 months | 0 (0.0) |

ADL: Activities of daily living.

IADL: Instrumental activities of daily living.

**Supplemental Table 6.** Frequency of self-reported gaps in care coordination overall and by frailty status.

| Gap in care coordination | Overall | Not frail | Intermediate-frail | Frail | P-trend |
| --- | --- | --- | --- | --- | --- |
|  | (n=5024) | (n=2398) | (n=2436) | (n=190) |  |
| In the past 6 months, the personal doctor only sometimes or never had medical records or other information about the respondent’s care when he or she had a scheduled appointment. | 144 (2.9) | 54 (2.3) | 81 (3.3) | 9 (4.7) | 0.008 |
| In the past 6 months, the personal doctor only sometimes or never talked about all of the prescription medicines the respondent was taking. | 803 (16.0) | 348 (14.5) | 416 (17.1) | 39 (20.5) | 0.004 |
| In the past 6 months, the personal doctor seemed informed and up-to-date about care the respondent received from specialists sometimes or never. | 400 (8.0) | 169 (7.1) | 213 (8.7) | 18 (9.5) | 0.021 |
| In the past 6 months, the respondent only somewhat received or did not receive the help he or she needed from the personal doctor’s office to manage his or her care among different providers and services. | 602 (12.0) | 241 (10.1) | 330 (13.6) | 31 (16.3) | <0.001 |
| In the past 6 months, the respondent’s personal doctor’s office followed up only sometimes or never to provide the results of a blood test, x-ray, or other test that the personal doctor had ordered/the patient only sometimes or never got those results as soon as they needed them. | 634 (12.6) | 262 (10.9) | 330 (13.6) | 42 (22.1) | <0.001 |
| The respondent thinks that, in general, the doctors he or she sees do not communicate with each other about his or her care. | 532 (10.6) | 248 (10.3) | 259 (10.6) | 25 (13.2) | 0.457 |
| The respondent rates the coordination of care among all of the health professionals that he or she sees, in general, as fair or poor. | 383 (7.6) | 169 (7.1) | 195 (8.0) | 19 (10.0) | 0.102 |
| Any of the above | 1979 (39.4) | 888 (37.0) | 994 (40.8) | 97 (51.1) | <0.001 |

**Supplemental Table 7.** Association between frailty and the number of questions to which participants reported gaps in care coordination.

|  | **Not frail** | **Intermediate-frail** | **Frail** | **P-trend** |
| --- | --- | --- | --- | --- |
| N | 2398 | 2436 | 190 |  |
| Number of questions reporting gaps in care coordination, n (%) |  |  |  |  |
| 0 | 1510 (63.0) | 1442 (59.2) | 93 (49.0) |  |
| 1 | 505 (21.1) | 525 (21.6) | 53 (27.9) | - |
| 2 | 235 (9.8) | 255 (10.5) | 20 (10.5) | - |
| 3 | 97 (4.1) | 119 (4.9) | 12 (6.3) | - |
| 4 | 36 (1.5) | 58 (2.4) | 8 (4.2) | - |
| ≥5 | 15 (0.6) | 37 (1.5) | 4 (2.1) | - |
| Mean (SD) | 0.62 (1.0) | 0.75 (1.2) | 0.96 (1.3) |  |
| Ratio of the mean number of questions reporting gaps in care coordination (95% confidence intervals)* | | | | |
| Model 1 (n=5,024) | 1 (Ref) | 1.20 (1.11 – 1.31) | 1.55 (1.29 – 1.87) | - |
| Model 2 (n=4,289) | 1 (Ref) | 1.17 (1.07 – 1.29) | 1.49 (1.21- 1.83) | - |
| Model 3 (n=3,713) | 1 (Ref) | 1.20 (1.09 – 1.33) | 1.67 (1.33 – 2.10) | - |
| Final model† (n=5,024) | 1 (Ref) | 1.19 (1.09 – 1.30) | 1.50 (1.23 – 1.82) | - |

Model 1 is unadjusted.

Model 2 includes adjustment for age, gender, race, education, annual household income, marital status, geographic region of residence, and rural area.

Model 3 includes adjustment for the variables in model 2 and hypertension, hyperlipidemia, diabetes, history of myocardial infarction, prior coronary revascularization, history of stroke, chronic kidney disease, atrial fibrillation, and self-rated health.

*Ratios were calculated using marginalized zero-inflated Poisson regression models.

†In the final model, multiple imputation was used to retain participants with missing data in covariates.

**Supplemental Table 8.** Sensitivity analyses for the association between frailty and any gap in care coordination.

|  | **Not frail** | **Intermediate-frail** | **Frail** | **P-trend** |
| --- | --- | --- | --- | --- |
| **Sensitivity analysis 1: removing weakness from the frailty definition* - panel A** | | | | |
| N | 2826 | 2125 | 73 |  |
| N (%) with ≥1 gap in care coordination | 1053 (37.3) | 884 (41.6) | 42 (57.5) | <0.001 |
| Prevalence ratios (95% confidence intervals) | | | | |
| Model 1 (n=5,024) | 1 (Ref) | 1.12 (1.04 – 1.20) | 1.54 (1.26 – 1.89) | <0.001 |
| Model 2 (n=4,289) | 1 (Ref) | 1.09 (1.01 – 1.18) | 1.56 (1.26 – 1.92) | 0.001 |
| Model 3 (n=3,713) | 1 (Ref) | 1.14 (1.05 – 1.23) | 1.71 (1.37 – 2.13) | <0.001 |
| Final model† (n=5,024) | 1 (Ref) | 1.11 (1.04 – 1.19) | 1.47 (1.20 – 1.81) | 0.001 |
| **Sensitivity analysis 2: removing history of falls from the frailty definition‡** - **panel B** | | | | |
| N | 2956 | 2024 | 44 |  |
| N (%) with ≥1 gap in care coordination | 1091 (36.9) | 866 (42.8) | 22 (50.0) | <0.001 |
| Prevalence ratios (95% confidence intervals) | | | | |
| Model 1 (n=5,024) | 1 (Ref) | 1.16 (1.08 – 1.24) | 1.35 (1.00 – 1.83) | <0.001 |
| Model 2 (n=4,289) | 1 (Ref) | 1.12 (1.04 – 1.20) | 1.37 (0.99 – 1.88) | 0.002 |
| Model 3 (n=3,713) | 1 (Ref) | 1.15 (1.06 – 1.25) | 1.39 (1.00 – 1.93) | 0.001 |
| Final model† (n=5,024) | 1 (Ref) | 1.15 (1.07 – 1.23) | 1.32 (0.98 – 1.78) | <0.001 |
| **Sensitivity analysis 3: adjusting for disability in ≥ 1 ADL and ≥ 1 IADL – panel C** | | | | |
| N | 2398 | 2436 | 190 |  |
| N (%) with ≥1 gap in care coordination | 888 (37.0) | 994 (40.8) | 97 (51.1) | <0.001 |
| Prevalence ratios (95% confidence intervals) | | | | |
| Model 1 (n=5,024) | 1 (Ref) | 1.10 (1.03 – 1.18) | 1.38 (1.19 – 1.60) | <0.001 |
| Model 2 (n=4,289) | 1 (Ref) | 1.03 (0.96 – 1.12) | 1.19 (1.01 – 1.41) | 0.113 |
| Model 3 (n=3,713) | 1 (Ref) | 1.07 (0.98 – 1.17) | 1.30 (1.09 – 1.57) | 0.013 |
| Final model† (n=5,024) | 1 (Ref) | 1.06 (0.99 – 1.15) | 1.25 (1.07 – 1.47) | 0.011 |
| **Sensitivity analysis 4: adjusting for social support**§ - **panel D** | | | | |
| N | 1848 | 1754 | 131 |  |
| N (%) with ≥1 gap in care coordination | 681 (36.9) | 705 (40.2) | 60 (45.8) | 0.011 |
| Prevalence ratios (95% confidence intervals) | | | | |
| Model 1 (n=3,733) | 1 (Ref) | 1.09 (1.00 – 1.18) | 1.24 (1.02 – 1.51) | 0.008 |
| Model 2 (n=3,172) | 1 (Ref) | 1.05 (0.96 – 1.15) | 1.11 (0.90 – 1.37) | 0.209 |
| Model 3 (n=2,754) | 1 (Ref) | 1.08 (0.98 – 1.19) | 1.23 (0.98 – 1.54) | 0.054 |
| Final model† (n=3,733) | 1 (Ref) | 1.07 (0.99 – 1.17) | 1.17 (0.96 – 1.43) | 0.043 |

Model 1 is unadjusted.

Model 2 includes adjustment for age, gender, race, education, annual household income, marital status, geographic region of residence, and rural area. For sensitivity analysis 3, model 2 also includes adjustment for ADL and IADL. For sensitivity analysis 4, model 2 also includes adjustment for social support.

Model 3 includes adjustment for the variables in model 2 and hypertension, hyperlipidemia, diabetes, history of myocardial infarction, prior coronary revascularization, history of stroke, chronic kidney disease, atrial fibrillation, and self-rated health.

*In sensitivity analysis 1, we defined frailty status using four indicators: Low body mass index, Exhaustion, Slow walk and History of falls (i.e., without weakness). In this sensitivity analysis, not frail was defined by having 0 indicators, intermediate-frailty was defined by having 1-2 indicators, and frailty was defined by having ≥ 3 out of the 4 indicators.

†In the final model, multiple imputation was used to retain participants with missing data in covariates.

‡In sensitivity analysis 2, we defined frailty status using four indicators: Low body mass index, Exhaustion, Slow walk and Weakness (i.e., without history of falls). In this sensitivity analysis, not frail was defined by having 0 indicators, intermediate-frailty was defined by having 1-2 indicators, and frailty was defined by having ≥ 3 out of the 4 indicators.

§Sensitivity analysis 4 was restricted to participants with data on social support.

ADL: activities of daily living.

IADL: instrumental activities of daily living.

**Supplemental Table 9**. Frequency of self-reported preventable adverse events in the previous 12 months by frailty status.

| Type of outcome experiences by the respondent over the previous 12 months | Number who responded yes (Numerator) / Denominator* (%) | | | |  |
| --- | --- | --- | --- | --- | --- |
|  | Overall | Not frail | Intermediate-frail | Frail | P-trend |
| Repeat test |  |  |  |  |  |
| Repeated a blood test, x-ray, or other test that the participant had had in the past 2 weeks because his or her doctor did not have the results of the first test (Outcome #1) | 144/5024 (2.9) | 50/2398 (2.1) | 80/2436 (3.3) | 14/190 (7.4) | <0.001 |
| Drug-drug interaction |  |  |  |  |  |
| Had a problem with his or her medications, because different doctors prescribed medications that did not go well together (Outcome #2) | 305/5024 (6.1) | 105/2398 (4.4) | 177/2436 (7.3) | 23/190 (12.1) | <0.001 |
| Emergency department visit |  |  |  |  |  |
| Has had one or more emergency department (ED) visits | 1226/5024 (24.4) | 505/2398 (21.1) | 648/2436 (26.6) | 73/190 (38.4) | <0.001 |
| Believes ED visit could have been prevented | 335/1226 (27.3) | 126/505 (25.0) | 194/648 (29.9) | 15/73 (20.5) | 0.294 |
| Believes ED visit could have been prevented by better communication across his or her doctors and other healthcare professionals (Outcome #3) | 73/335 (21.8) | 25/126 (19.8) | 39/194 (20.1) | 9/15 (60.0) | 0.118 |
| Hospital admission |  |  |  |  |  |
| Has had one or more hospital admissions | 1005/5024 (20.0) | 418/2398 (17.4) | 526/2436 (21.6) | 61/190 (32.1) | <0.001 |
| Believes hospital admission could have been prevented | 147/1005 (14.6) | 67/418 (16.0) | 70/526 (13.3) | 10/61 (16.4) | 0.395 |
| Believes hospital admission could have been prevented by better communication across his or her doctors and other healthcare professionals (outcome #4) | 36/147 (24.5) | 12/67 (17.9) | 21/70 (30.0) | 3/10 (30.0) | 0.102 |
| Any of the four outcomes |  |  |  |  |  |
| Any of outcome #1, #2, #3, #4 | 481/5024 (9.6) | 167/2398 (7.0) | 276/2436 (11.3) | 38/190 (20.0) | <0.001 |

*The denominator varies due to the skip logic embedded in the survey.

**Supplemental** **Table 10.** Sensitivity analyses for the association between frailty and any preventable adverse event.

|  | **Not frail** | **Intermediate-frail** | **Frail** | **P-trend** |
| --- | --- | --- | --- | --- |
| **Sensitivity analysis 1: removing weakness from the frailty definition*** | | | | |
| N | 2826 | 2125 | 73 |  |
| Prevalence, % | 207 (7.3) | 258 (12.1) | 16 (21.9) | <0.001 |
| Prevalence ratios (95% confidence intervals) | | | | |
| Model 1 (n=5,024) | 1 (Ref) | 1.66 (1.39 – 1.97) | 2.99 (1.90 – 4.70) | <0.001 |
| Model 2 (n=4,289) | 1 (Ref) | 1.65 (1.37 – 2.00) | 3.18 (1.95 – 5.17) | <0.001 |
| Model 3 (n=3,713) | 1 (Ref) | 1.61 (1.30 – 1.98) | 2.93 (1.65 – 5.21) | <0.001 |
| Final model† (n=5,024) | 1 (Ref) | 1.52 (1.27 – 1.81) | 2.24 (1.37 – 3.66) | <0.001 |
| **Sensitivity analysis 2: removing history of falls from the frailty definition‡** | | | | |
| N | 2956 | 2024 | 44 |  |
| Prevalence, % | 224 (7.6) | 248 (12.3) | 9 (20.5) | <0.001 |
| Prevalence ratios (95% confidence intervals) | | | | |
| Model 1 (n=5,024) | 1 (Ref) | 1.62 (1.36 – 1.92) | 2.70 (1.49 – 4.90) | <0.001 |
| Model 2 (n=4,289) | 1 (Ref) | 1.60 (1.32 – 1.93) | 2.01 (0.93 – 4.33) | <0.001 |
| Model 3 (n=3,713) | 1 (Ref) | 1.50 (1.21 – 1.85) | 1.91 (0.87 – 4.19) | <0.001 |
| Final model† (n=5,024) | 1 (Ref) | 1.41 (1.18 – 1.68) | 1.96 (1.03 – 3.70) | <0.001 |
| **Sensitivity analysis 3: adjusting for disability in ≥ 1 ADL and ≥ 1 IADL** | | | | |
| N | 2398 | 2436 | 190 |  |
| Prevalence, % | 167 (7.0) | 276 (11.3) | 38 (20.0) | <0.001 |
| Risk ratios (95% confidence intervals) | | | | |
| Model 1 (n=5,024) | 1 (Ref) | 1.63 (1.35 – 1.96) | 2.87 (2.09 – 3.95) | <0.001 |
| Model 2 (n=4,289) | 1 (Ref) | 1.53 (1.24 – 1.88) | 2.14 (1.46 – 3.12) | <0.001 |
| Model 3 (n=3,713) | 1 (Ref) | 1.50 (1.19 – 1.89) | 2.03 (1.34 – 3.08) | <0.001 |
| Final model† (n=5,024) | 1 (Ref) | 1.39 (1.15 – 1.68) | 1.97 (1.39 – 2.78) | <0.001 |
| **Sensitivity analysis 4: adjusting for social support§** | | | | |
| N | 1848 | 1754 | 131 |  |
| Prevalence, % | 116 (6.3) | 184 (10.5) | 25 (19.1) | <0.001 |
| Prevalence ratios (95% confidence intervals) | | | | |
| Model 1 (n=3,733) | 1 (Ref) | 1.67 (1.34 – 2.09) | 3.04 (2.05 – 4.51) | <0.001 |
| Model 2 (n=3,172) | 1 (Ref) | 1.73 (1.35 – 2.21) | 2.57 (1.63 – 4.06) | <0.001 |
| Model 3 (n=2,754) | 1 (Ref) | 1.65 (1.26 – 2.17) | 2.18 (1.27 – 3.76) | <0.001 |
| Final model† (n=3,733) | 1 (Ref) | 1.50 (1.20 – 1.89) | 2.12 (1.39 – 3.22) | <0.001 |

Model 1 is unadjusted.

Model 2 includes adjustment for age, gender, race, education, annual household income, marital status, geographic region of residence and rural area. For sensitivity analysis 3, model 2 also includes adjustment for ADL and IADL. For sensitivity analysis 4, model 2 also includes adjustment for social support.

Model 3 includes adjustment for the variables in model 2 and hypertension, hyperlipidemia, diabetes, history of myocardial infarction, prior coronary revascularization, history of stroke, chronic kidney disease, atrial fibrillation, and self-rated health.

*In sensitivity analysis 1, we defined frailty status using four indicators: Low body mass index, Exhaustion, Slow walk and History of falls (i.e., without weakness). In this sensitivity analysis, not frail was defined by having 0 indicators, intermediate-frailty was defined by having 1-2 indicators, and frailty was defined by having ≥ 3 out of the 4 indicators.

†In the final model, multiple imputation was used to retain participants with missing data in covariates.

‡In sensitivity analysis 2, we defined frailty status using four indicators: Low body mass index, Exhaustion, Slow walk and Weakness (i.e., without history of falls). In this sensitivity analysis, not frail was defined by having 0 indicators, intermediate-frailty was defined by having 1-2 indicators, and frailty was defined by having ≥ 3 out of the 4 indicators.

§Sensitivity analysis 3 was restricted to participants with data on social support.

ADL: activities of daily living.

IADL: instrumental activities of daily living.

**Supplemental Table 11.** Sensitivity analyses for the association between any gap in care coordination and any preventable adverse events, among participants with intermediate-frailty or frailty.

|  | No gap in care coordination | Gap in care coordination |
| --- | --- | --- |
| **Sensitivity analysis 1: removing weakness from the frailty definition*** | | |
| N | 1272 | 926 |
| Prevalence of any preventable adverse outcome, % | 136 (10.7) | 138 (14.9) |
| Prevalence ratios (95% confidence intervals) for any preventable adverse outcome | | |
| Model 1 (n=2,198) | 1 (Ref) | 1.39 (1.12 – 1.74) |
| Model 2 (n=1,864) | 1 (Ref) | 1.26 (0.99 – 1.60) |
| Model 3 (n=1,583) | 1 (Ref) | 1.30 (1.00 – 1.69) |
| Final model† (n=2,198) | 1 (Ref) | 1.37 (1.10 – 1.71) |
| **Sensitivity analysis 2: removing history of falls from the frailty definition‡** | | |
| N | 1180 | 888 |
| Prevalence of any preventable adverse outcome, % | 126 (10.7) | 131 (14.8) |
| Prevalence ratios (95% confidence intervals) for any preventable adverse outcome | | |
| Model 1 (n=2,068) | 1 (Ref) | 1.38 (1.10 – 1.74) |
| Model 2 (n=1,747) | 1 (Ref) | 1.26 (0.98 – 1.62) |
| Model 3 (n=1,468) | 1 (Ref) | 1.36 (1.03 – 1.79) |
| Final model† (n=2,068) | 1 (Ref) | 1.37 (1.09 – 1.71) |
| **Sensitivity analysis 3: adjusting for disability in ≥ 1 ADL and ≥ 1 IADL** | | |
| N | 1535 | 1091 |
| Prevalence of any preventable adverse outcome, % | 154 (10.0) | 160 (14.7) |
| Prevalence ratios (95% confidence intervals) for any preventable adverse outcome | | |
| Model 1 (n=2,626) | 1 (Ref) | 1.46 (1.19 – 1.80) |
| Model 2 (n=2,234) | 1 (Ref) | 1.28 (1.02 – 1.60) |
| Model 3 (n=1,892) | 1 (Ref) | 1.32 (1.03 – 1.69) |
| Final model† (n=2,626) | 1 (Ref) | 1.41 (1.14 – 1.74) |
| **Sensitivity analysis 4: adjusting for social support§** | | |
| N | 1120 | 765 |
| Prevalence of any preventable adverse outcome, % | 107 (9.6) | 102 (13.3) |
| Prevalence ratios (95% confidence intervals) for any preventable adverse outcome | | |
| Model 1 (n=1,885) | 1 (Ref) | 1.40 (1.08 – 1.80) |
| Model 2 (n=1,602) | 1 (Ref) | 1.29 (0.98 – 1.70) |
| Model 3 (n=1,353) | 1 (Ref) | 1.35 (0.99 – 1.85) |
| Final model† (1,885) | 1 (Ref) | 1.38 (1.07 – 1.78) |

Model 1 is unadjusted

Model 2 includes adjustment for age, gender, race, education, annual household income, marital status, geographic region of residence and rural area. For sensitivity analysis 3, model 2 also includes adjustment for ADL and IADL. For sensitivity analysis 4, model 2 also includes adjustment for social support.

Model 3 includes adjustment for the variables in model 2 and hypertension, hyperlipidemia, diabetes, history of myocardial infarction, prior coronary revascularization, history of stroke, chronic kidney disease, atrial fibrillation, and self-rated health.

*In sensitivity analysis 1, we defined frailty status using four indicators: Low body mass index, Exhaustion, Slow walk and History of falls (i.e., without weakness). In this sensitivity analysis, not frail was defined by having 0 indicators, intermediate-frailty was defined by having 1-2 indicators, and frailty was defined by having ≥ 3 out of the 4 indicators.

†In the final model, multiple imputation was used to retain participants with missing data in covariates.

‡In sensitivity analysis 2, we defined frailty status using four indicators: Low body mass index, Exhaustion, Slow walk and Weakness (i.e., without history of falls). In this sensitivity analysis, not frail was defined by having 0 indicators, intermediate-frailty was defined by having 1-2 indicators, and frailty was defined by having ≥ 3 out of the 4 indicators.

§Sensitivity analysis 4 was restricted to participants with data on social support.

ADL: activities of daily living.

IADL: instrumental activities of daily living.

**Supplemental Figure 1.** Flow-chart of REGARDS study participants included in the current analysis.

| **30,239** completed the 1^st^ REGARDS CATI and in-home examination in 2003-2007 |
| --- |
|  |
|  |
|  |
|  |
|  |
| **14,236** completed the 2^nd^ REGARDS CATI and in-home examination in 2013-2016 |
|  |
|  |
| **2,308** excluded because were <65 years at the REGARDS 2^nd^ in-home examination |
|  |
| **11,928** aged ≥ 65 years at the time of the REGARDS 2^nd^ in-home examination |
|  |
| **16,003** excluded because did not complete the REGARDS 2^nd^ CATI and in-home examination |
|  |
| **2,503** excluded because did not complete the healthcare survey module |
|  |
|  |
| **9,425** completed the healthcare survey in 2017-2018 |
|  |
|  |
| **1,879** excluded because did not have a regular healthcare provider or did not see the provider in the last 6 months |
|  |
|  |
|  |
| **7,546** had a regular healthcare provider and saw the provider in the last 6 months |
|  |
|  |
| **2,028** excluded because reported having ≤ 1 visit or ≤ 1 provider in the previous year |
|  |
|  |
| **5,518** reported > 1 visit and > 1 provider in the previous year |
|  |
|  |
| **479** excluded because had cognitive impairment* |
|  |
|  |
| **5,039** without cognitive impairment* |
|  |
|  |
|  |
| **15** excluded without valid data on ≥3 indicators for frailty |
|  |
|  |
| **5,024** with valid data for at least 3 out of the 5 indicators for frailty |
|  |
|  |

REGARDS: REasons for Geographic And Racial Differences in Stroke.

CATI: Computer Assisted Telephone Interview.

* Cognitive impairment was defined as having a Six-Item Screener score ≤4 in the last assessment on or before the completion of the survey module on experiences with healthcare, or by having 2 consecutive Six-Item Screener scores ≤4 any time on or before the completion of the survey module on experiences with healthcare. The Six-Item Screener is administered biannually in the REGARDS study.

**Supplemental Figure 2**. Timing of data collection for variables used in this analysis.

**Baseline CATI and in-home study visit: 2003-2007**

- Gender
- Race
- Educational attainment
- Region of residence
- Rural area
- Self-reported stroke history

**Survey on experiences with healthcare: 2017-2018**

- Gaps in care coordination
- Preventable adverse events
- Number of ambulatory visits*
- Number of ambulatory providers*

**Second CATI and in-home study visit: 2013-2016**

- Low body mass index
- Exhaustion: CESD scale
- Slow walk
- Weakness: Chair stand test
- History of falls
- Age
- Annual household income
- Marital status
- Hypertension
- Dyslipidemia
- Diabetes
- Prior coronary revascularization
- Chronic kidney disease
- Self-reported history of atrial fibrillation and on the study electrocardiogram
- Self-reported history of myocardial infarction
- Self-rated health
- Social support
- Activities of daily living
- Instrumental activities of daily living

CATI: Computer-assisted telephone interview; CESD: Center for Epidemiologic Studies Depression; REGARDS: REasons for Geographic And Racial Differences in Stroke Study.

* In the 12 months preceding the survey on experiences with healthcare.

**Follow-up for cardiovascular events**

- Adjudicated myocardial infarctions
- Adjudicated stroke events

**References for the supplemental materials**

1. Fried LP, Tangen CM, Walston J, et al. Frailty in older adults: evidence for a phenotype. *J Gerontol A Biol Sci Med Sci.* 2001;56(3):M146-156.

2. Radloff LS. The CES-D Scale:A Self-Report Depression Scale for Research in the General Population. *Applied Psychological Measurement.* 1977;1(3):385-401.

3. Kiely DK, Cupples LA, Lipsitz LA. Validation and comparison of two frailty indexes: The MOBILIZE Boston Study. *Journal of the American Geriatrics Society.* 2009;57(9):1532-1539.

4. Kern LM, Reshetnyak E, Colantonio LD, et al. Association Between Patients' Self-Reported Gaps in Care Coordination and Preventable Adverse Outcomes: a Cross-Sectional Survey. *J Gen Intern Med.* 2020;35(12):3517-3524.

5. Hays RD, Martino S, Brown JA, et al. Evaluation of a Care Coordination Measure for the Consumer Assessment of Healthcare Providers and Systems (CAHPS) Medicare survey. *Med Care Res Rev.* 2014;71(2):192-202.

6. Safford MM, Brown TM, Muntner PM, et al. Association of race and sex with risk of incident acute coronary heart disease events. *Jama.* 2012;308(17):1768-1774.

7. Howard VJ, Kleindorfer DO, Judd SE, et al. Disparities in stroke incidence contributing to disparities in stroke mortality. *Ann Neurol.* 2011;69(4):619-627.
